# Regenerating myofiber with activating of TGF-β signaling contributes to macrophage efferocytosis through enhancing Tregs response in inflamed muscle

**DOI:** 10.3389/fimmu.2026.1810106

**Published:** 2026-04-22

**Authors:** HaiQiang Lan, XiaoTing Jian, ZhaoHong Liao, YangYang Li, QiSen Wang, JingWen Huang, JiJie Hu, Hua Liao

**Affiliations:** 1 Guangdong Provincial Key Laboratory of Construction and Detection in Tissue Engineering; Department of Anatomy, School of Basic Medical Sciences, Southern Medical University, Guangzhou, China; 2 Department of Laboratory Medicine, School of Medicine, Foshan University, Foshan, China; 3 Department of Orthopaedics and Traumatology, Nanfang Hospital, Southern Medical University, Guangzhou, China

**Keywords:** IL-6, macrophage efferocytosis, muscle injury, TGF-β signaling, Tregs

## Abstract

**Background:**

This study aimed to investigate the role of myofiber-specific TGF-β signaling in the development of muscle inflammation by modulating Treg-cell-mediated macrophage efferocytosis.

**Methods:**

CTX-induced muscle injury was performed in the tibialis anterior (TA) of control (TGF-βr2^flox/flox^) and transgenic mice with skeletal muscle-specific deletion of TGF-β receptor 2 (SM TGF-βr2^−/−^). Gene levels of regulatory T cell (Treg) activation markers and inflammatory mediators produced by macrophages or Tregs were assessed using qRT-PCR. Intramuscular infiltration of Tregs and macrophages, as well macrophage phenotypes, efferocytic function, and associated signaling molecules, were evaluated using hematoxylin and eosin (HE) staining, immunofluorescence, immunoblotting and FACS analysis. The correlation of myofibers with Tregs-mediated macrophage efferocytosis were addressed under an *in vitro* co-culture system, which including Tregs, macrophages, and the differentiated myogenic precursor cells (MPC-myotubes) isolated from control or SM TGF-βr2^−/−^mice. Apoptotic cells were generated by UV irradiation prior to transfer into inflamed muscle.

**Results:**

Deficiency in muscle TGF-β signaling resulted in more severe muscle inflammation, characterized by an increased number of M1 macrophages and a decreased number of M2 macrophages. Notably, the absence of muscle TGF-β signaling impaired the efferocytic capacity of macrophages and reduced the proportion of Tregs in inflamed muscle. Further, we monitored that activation of intrinsic TGF-β signaling suppresses myofiber IL-6 production, which in turn impacted on IL-13 production from Tregs accumulated in damaged muscle. This ultimately facilitates IL-10-STAT3-Vav1-mediated macrophage efferocytosis in inflamed muscle.

**Conclusions:**

Our findings establish a link between muscle-specific TGF-β signaling, myokine IL-6, Tregs derived IL-13 and macrophage efferocytosis in inflamed muscle. These results suggest that therapeutic targeting of this axis may hold promise for promoting muscle regeneration.

## 1 Introduction

Efferocytosis, the phagocytosis of apoptotic cells, is a critical process that promotes the shift of macrophages toward an anti-inflammatory phenotype and facilitates the resolution of inflammation, thereby enabling tissue regeneration (1, 2). In the context of muscle inflammation induced by inflammatory myopathies (3–5), excessive exercise (6–9), or acute injury (10), apoptosis occurs in various cell types, including myoblasts, multinucleated myofibers, fibro-adipogenic progenitors (FAPs), stromal cells, and infiltrating leukocytes. Macrophage-mediated efferocytosis contributes to the resolution of muscle inflammation by promoting an anti-inflammatory/reparative phenotype, which dampens inflammatory responses and supports myofiber repair, angiogenesis, and extracellular matrix remodeling (11, 12). Accordingly, defective macrophage efferocytosis is associated with impaired muscle regeneration due to a failure in the phenotypic transition of macrophages toward a reparative state *in vivo* (12).

Following muscle injury, the peak accumulation of anti-inflammatory macrophages closely coincides with the influx of regulatory T cells (Tregs) into inflamed muscle. Tregs rapidly expand during the period when macrophages transition from a pro-inflammatory (Ly6C^+^ M1) to an anti-inflammatory (Ly6C^-^ M2) phenotype, suggesting that Tregs play a role in facilitating this phenotypic switch, a process essential for effective muscle repair (13, 14). Notably, Tregs have been shown to enhance macrophage efferocytosis *via* a transcellular signaling mechanism, in which Treg-derived IL-13 activates an IL-10-Vav1-Rac1 pathway that promotes the internalization of apoptotic cells by macrophages (15). Although Tregs-mediated regulation is critical for the inflammation-repair transition in skeletal muscle, the involvement of Tregs-dependent macrophage efferocytosis in the context of muscle injury has not yet been elucidated.

During muscle inflammation and remodeling, transforming growth factor-beta (TGF-β) promotes the proliferation, differentiation, and fusion of satellite cells into multinucleated myofibers (16, 17). In parallel, TGF-β serves as a key initiator of extracellular matrix (ECM) deposition and tissue fibrosis (18, 19). Emerging evidence indicates that TGF-β signaling also modulates muscle immune function by directly regulating the expression of inflammatory mediators in myofibers, such as intercellular adhesion molecule-1 (ICAM-1), human leukocyte antigen class I (HLA I), and interleukin-6 (IL-6) (20–22). Our recent work demonstrated that activation of TGF-β signaling specifically in muscle cells can suppress local inflammation by inhibiting Th17 responses and blocking macrophage transition, while concurrently promoting Tregs response (23). These observations raise the question: Does myofiber-specific TGF-β signaling contribute to the regulation of local inflammation after muscle injury by modulating Tregs-mediated macrophage efferocytosis?

The present study was designed to address this question. Our findings demonstrate that deficiency of muscle-specific TGF-β signaling reduces the accumulation of M2 macrophages, impairs macrophage efferocytic capacity, and decreases the proportion of Tregs in inflamed muscle. Furthermore, our data suggest a role for myofibers in promoting the resolution of muscle inflammation and facilitating tissue repair, through intrinsic TGF-β signaling, which directs Tregs-mediated macrophage efferocytosis in inflamed microenvironment.

## 2 Materials and methods

### 2.1 Ethical approval

All animal experiments were approved by the Animal Experimentation Ethics Committee of Southern Medical University (Approval No. L2016068).

### 2.2 Mouse strains and animal experiments

C57BL/6 (B6) mice (6-8 weeks) were obtained from the Animal Experimentation Centre of Southern Medical University. Skeletal muscle-specific TGF-β receptor 2 knockout mice (referred to as SM TGF-βr2^−/−^) were generated by crossing MCK-Cre mice (The Jackson Laboratory) with floxed TGF-βr2 mice (TGF-βr2^flox/flox^, The Jackson Laboratory). Genotypes of the resulting offspring were confirmed by polymerase chain reaction (PCR) using DNA extracted from tail samples. TGF-βr2^flox/flox^ mice served as controls for SM TGF-βr2^−/−^ mice.

An acute muscle injury model was established by injecting 60 μL of Cardiotoxin (CTX) solution (50 μg/mL; TX4001, Orientoxin, China) into the unilateral tibialis anterior (TA) muscles of male mice aged 6-8 weeks. Sham-operated mice received PBS injections. All animals were maintained under a 12-hour light/dark cycle and were humanely euthanized by cervical dislocation on day 3, 7, 10, or 15 post-injury. Muscle samples were collected and immediately frozen for subsequent gene and protein analyses. For histological examination, muscle tissues were flash-frozen in isopentane cooled with liquid nitrogen.

To assess the uptake of exogenous apoptotic cells by macrophages in inflamed muscle, neutrophils were isolated from the spleen of B6 mice using density gradient centrifugation with Ficoll Plus 1.083 (P4360, Solarbio, China). Apoptosis was induced by UV irradiation (254 nm, 30 minutes), and apoptotic cells were labeled with the membrane-intercalating dye DiD (C1039) or assessed by TUNEL staining (40306ES60, Yeasen, China). A total of 1×10^6^ apoptotic cells were injected into the injured TA muscle 12 hours prior to muscle sample collection.

### 2.3 Tregs depletion and transfer

For removing Tregs *in vivo*, wild B6 mice received intraperitoneal injections of anti-CD25 antibody (PC61,500ug) (25-0251-82, eBioscience™, USA), a commonly used method for depleting Treg cells.

For Tregs transfer, CD4^+^ cells were isolated from the spleen of B6 mice by density gradient centrifugation, and CD4^+^ CD25^+^ Tregs were subsequently sorted by flow cytometry. Purified Tregs (1×10^6^) were then transferred into the inflamed TA muscles of both SM TGF-βr2^−/−^ and control mice on day 1 following CTX-treatment.

### 2.4 Primary cell cultures and pro-inflammatory stimulation

Murine myogenic precursor cells (MPCs) were isolated from the limb muscles of neonatal control or SM TGF-βr2^−/−^ mice. Muscle tissue was digested with Collagenase II (V900892-1G, Sigma, USA), filtered, and centrifuged to obtain a single-cell suspension. MPCs were purified using the Mice Satellite Cell Isolation Kit (130-104-268, Miltenyi Biotec, Cologne, Germany). Briefly, isolated cells were resuspended, treated with Enzyme A, incubated, and then mixed with Satellite Cell Isolation solution. The cell suspension was passed through an LS column in a MACS Separator (Miltenyi Biotec), and the flow-through containing unlabeled satellite cells was collected. Cells were cultured in DMEM/F12 (SH30023.01B, HyClone, Logan, UT, USA) supplemented with 10% fetal bovine serum (FBS, 10270106, Gibco, USA) and penicillin-streptomycin (100 μg/mL, SV30010, HyClone, USA). Upon reaching 70-80% confluence, the growth medium was replaced with differentiation medium containing 2% horse serum (HS) for 72 hours to induce differentiation into myotubes (MPC-Myotubes). For pro-inflammatory stimulation, MPC-Myotubes were treated with lipopolysaccharide (LPS, 100 ng/mL, L8880, Solarbio, China) and IFN-γ (3 ng/mL, 485-MI-100, R&D, USA).

Peritoneal macrophages were harvested by injecting 2 mL of the complete thioglycollate medium (146220, Sigma, USA) into the peritoneal cavity of wild B6 mice. After 72 hours, mice were euthanized, and macrophages were isolated from peritoneal lavage fluid. Cells were cultured in DMEM with 10% FBS and penicillin-streptomycin. For M2 polarization, macrophages were stimulated with IL-4 (100 ng/mL, 51084-MNAE, Sino Biological, China).

Tregs were isolated from the spleen of B6 mice and sorted by flow cytometry as CD4^+^CD25^+^ cells. Isolated Tregs were cultured in RPMI 1640 medium supplemented with IL-2 (10 ng/mL, 550069, BD Pharmingen™, USA), anti-CD3 (5 μg/mL, 553057, BD Pharmingen™, USA), and anti-CD28 (1 μg/mL, 553294, BD Pharmingen™, USA).

### 2.5 *In vitro* cell co-culture model and efferocytosis analysis

MPC-Myotubes derived from control or SM TGF-βr2^−/−^ mice were differentiated with horse serum for 72 hours and then treated with LPS and IFN-γ for 24 hours. Subsequently, they were co-cultured with Tregs and peritoneal macrophages for 8 hours at a ratio of 1:2:4 (MPC-Myotubes: Tregs:macrophages). Macrophages were then isolated and incubated with labeled apoptotic cells (3×10^6^ cells) for 45 minutes prior to efferocytosis assays. To re-activate TGF-β/Smad signaling in TGF-βr2-deficient MPC-Myotubes, cells were treated with the Smad agonist SRI-011381 hydrochloride (SRI, 8 μg/mL, HY-100347A, MedChemExpress, USA) before co-culture. An IL-6 inhibitor (AH, HY-N7674A, MedChemExpress, USA) was added to the culture media during co-culture to evaluate the effect of IL-6 on cellular responses. All chemical treatments were applied approximately 4 hours before efferocytosis assays.

### 2.6 Quantitative real-time PCR analysis

Total RNA was extracted from cultured MPC-Myotubes, muscle tissue samples, FACS-sorted Tregs or macrophages using TRIzol reagent (15596026, Invitrogen, USA). RNA (1μg) was reverse-transcribed into cDNA using the RevertAid First Strand cDNA Synthesis Kit (K1622, Thermo Scientific™, USA). Real-time quantitative PCR (qRT-PCR) was performed using SYBR Green/ROX qPCR Master Mix (A313-10, Genstar, China) with specific primers on an ABI StepOnePlus system (Sangon, China). Relative mRNA levels of ICOS, CTLA-4, Arg1, Retnla, Mrc1, iNOS, IL-6, TNF-α, IL-10, Bcl-3, and IL-13 were measured. Glyceraldehyde-3-phosphate dehydrogenase (GAPDH) was used as an endogenous control. Relative gene expression was calculated using the ΔΔCt method, and fold changes were presented as “2−ΔΔCt”.

### 2.7 Histological and immunofluorescence analysis

Snap-frozen TA muscles were transversely cryosectioned (8 μm), fixed in cold acetone, permeabilized with 0.1% Triton X-100 (P0096, Beyotime, China), and stained with hematoxylin and eosin (HE) or processed for immunofluorescence. For *in vitro* immunolabeling, cultured cells were fixed in 4% paraformaldehyde (P0099, Beyotime, China) for 20 minutes, permeabilized with 0.1% Triton X-100, and washed twice in PBS. The following primary antibodies were used: rat anti-mouse F4/80 (12-4801-82, Invitrogen, USA), rat anti-mouse CD11b (14-0112-85, eBioscience, USA), rabbit polyclonal anti-dystrophin (12715-1-AP, Proteintech, USA), rabbit anti-p-STAT3 (PA5-85445, Invitrogen, USA), rabbit polyclonal anti-CD3ε (ab16669, Abcam, USA), rabbit monoclonal anti-CD4 (14-0041-82, Thermo Fisher, USA), and anti-TUNEL-FITC (40306ES60, Yeasen, China). Alexa Fluor 488-conjugated goat anti-rabbit IgG (A0423), 555-conjugated donkey anti-rabbit IgG (A0453), 555-conjugated donkey anti-mouse IgG (A0460), or Cy3-conjugated goat anti-rat IgG (A0507) were used as secondary antibodies (Beyotime, China). Nuclei were counterstained with DAPI (ab104139, Abcam, UK). Slides and cell coverslips were examined under an Olympus BX51 fluorescence microscope (Olympus, Tokyo, Japan). Staining intensity was quantified using Image-Pro-Plus software from three independent experiments at 20× magnification.

### 2.8 Cell sorting and FACS analysis

Muscle samples were collected, minced, and digested with 0.2% type II collagenase (Sigma, USA) at 37°C for 40 minutes. Total cells were isolated from muscle homogenates and blocked. For *in vitro* cultures, cells were digested with trypsin (Sigma, USA) and resuspended in FACS buffer (PBS, 0.5% bovine serum albumin, 2 mM EDTA) to obtain single-cell suspensions. The following fluorescent antibodies were used: anti-CD3-APC (17-0032-82, Thermo Fisher, USA), anti-CD4-FITC (11-0041-85, Thermo Fisher, USA), anti-F4/80-PE (12-4801-82, Thermo Fisher, USA), anti-CD11b-FITC (11-0112-85, Thermo Fisher, USA), anti-Ly6C-Alexa Fluor™ 488 (53-5932-82, Thermo Fisher, USA), anti-CD206-PerCP-eFluor™ 710 (56-2061-82, Thermo Fisher, USA), anti-TUNEL-FITC (40306ES60, Yeasen, China), anti-Annexin-V-APC (E-CK-A117, Elabscience, USA), anti-CD25-APC (12-0251-82, Thermo Fisher, USA), anti-CD25-PE-Cyanine7 (25-0251-82, Thermo Fisher, USA), anti-GP130-PE (12-1302-82, Thermo Fisher, USA), anti-GP130-APC (17-1302-82, Thermo Fisher, USA), DiD (C1039, Beyotime, China), anti-FOXP3-APC (17-5773-82, Thermo Fisher, USA), anti-IL-13-eFluor 450 (48-7133-82, eBioscience, USA), anti-IL-10-Alexa Fluor 700 (56-7101-82, eBioscience, USA), anti-Bcl3-FITC (MA5-17667, Thermo Fisher, USA), anti-p-STAT3-eFluor 450 (48-9033-42, Thermo Fisher, USA), anti-Vav1-FITC (orb7274, Biorbyt, USA), and anti-Rac1-GTP-488 (CL488-66122, Proteintech, USA). Labeled cells were analyzed using a FACSAria II cell sorter with FlowJo software (BD Biosciences, USA).

### 2.9 Western blot analysis

Cellular or tissue proteins were extracted using a protein extraction kit (KGB5303, KeyGEN, China). The following antibodies were used for detection: rat anti-mouse IL-6 (AMC0864, Invitrogen, USA), mouse anti-mouse p-STAT3, and mouse anti-mouse GAPDH (AP0063, Bioworld, USA). Membranes were washed three times with TBST and then incubated with HRP-conjugated anti-rabbit IgG (FDR007, Fudebio, China) or anti-mouse IgG (FDM007, Fudebio, China) secondary antibodies. Protein bands were visualized using an ECL detection system (FD8001, Fudebio, China) and analyzed with ImageJ v1.42 software (National Institutes of Health, Bethesda, MD, USA).

### 2.10 Statistical analysis

Quantitative data are expressed as mean ± standard deviation. Statistical analyses were performed using SPSS version 20.0 software (IBM, Armonk, NY, USA). One-way ANOVA or two-sample *t*-tests were used for multiple or independent comparisons, respectively. A *p*-value < 0.05 was considered statistically significant.

## 3 Results

### 3.1 Deficiency of muscle TGF-β signaling promotes macrophage and CD4^+^ T cell accumulation but downregulates the Tregs ratio in inflamed muscle following CTX injection

**Figure 1:**
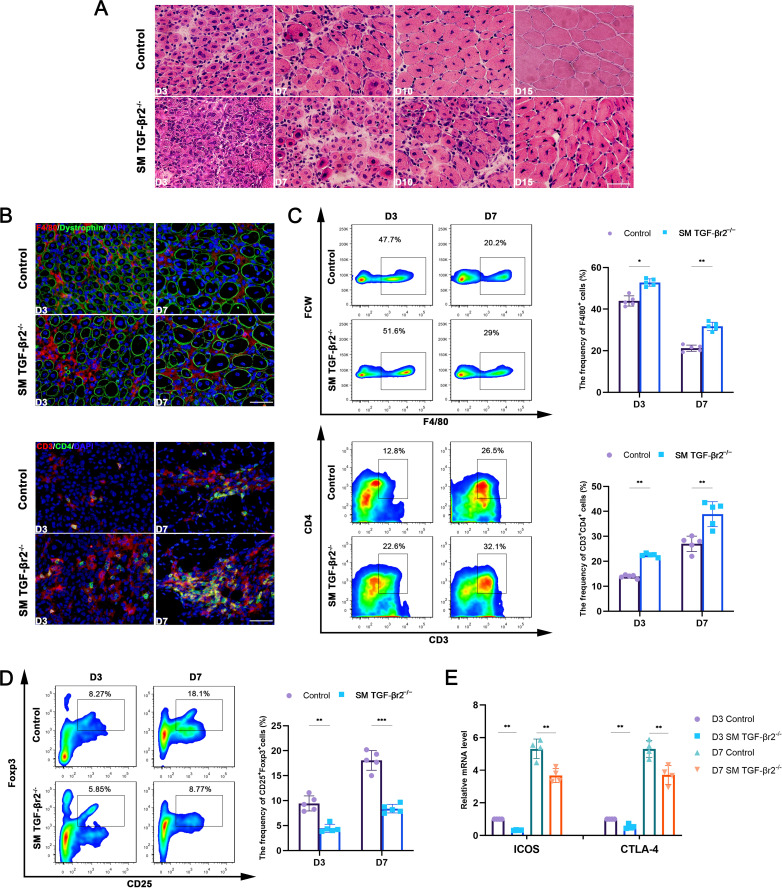
Deficiency of muscle TGF-β signaling exacerbates muscle inflammation. **(A)** H&E staining of inflamed TA muscle after CTX treatment. **(B)** Immunofluorescence staining of F4/80, Dystrophin, CD3 and CD4 on inflamed muscle. **(C)** FACS analysis of the proportion of F4/80^+^ and CD3^+^CD4^+^ cells. **(D)** FACS analysis of the proportion of CD25^+^Foxp3^+^ cells. **(E)** qRT-PCR analysis of gene levels of CTLA-4 and ICOS in CD4^+^ T cells sorted from inflamed muscle. Multiple comparisons are analyzed by One-way ANOVA. Data presented as mean ± SD (*n* = 4-5 mice per group). *p < 0.05, **p < 0.01, ***p < 0.001. Scale bar=50 μm.

To investigate the role of muscle-specific TGF-β signaling in muscle inflammation and myofiber immune function, we generated SM TGF-βr2^−/−^ mice resulting in targeted blockade of myofiber TGF-β signaling (23). We first re-evaluated the inflammatory features of CTX-injured TA muscles lacking TGF-β signaling. Consistent with our previous report (23), we observed a more severe inflammatory response in the inflamed muscles of SM TGF-βr2^−/−^ mice on day 3, 7, 10, and 15 post-injury compared to the control TGF-βr2^flox/flox^ mice (**Figure 1A**). Immunostaining and FACS analysis further revealed that deficiency of muscle TGF-β signaling led to increased infiltration of macrophages (F4/80^+^) and CD4^+^ T cells (CD3ε^+^CD4^+^) during the degenerative stage (day 3) and early regenerative stage (day 7) after injury (**Figures 1B, C**).

Given the central role of Foxp3^+^ regulatory T cells (Tregs) in resolving muscle inflammation (14, 24) and the essential function of TGF-β signaling in Tregs differentiation and activity (25), we next examined the frequency of Tregs among infiltrating CD4^+^ T cells. Notably, although the total number of CD4^+^ T cells was higher in inflamed muscles lacking TGF-β signaling, the percentage of CD4^+^CD25^+^Foxp3^+^ Tregs was markedly reduced compared with that in control mice (**Figure 1D**). Accordingly, gene expression levels of the Treg activation markers CTLA-4 and ICOS were significantly lower in CD4^+^ T cells sorted from inflamed TGF-βr2^−/−^ muscle than in those from control muscle (**Figure 1E**). These findings reveal a specific role for muscle-intrinsic TGF-β signaling in regulating macrophage and Treg responses in injured and inflamed muscle.

### 3.2 Muscle-specific TGF-β signaling affects the efferocytic capacity of macrophages in inflamed muscle

**Figure 2:**
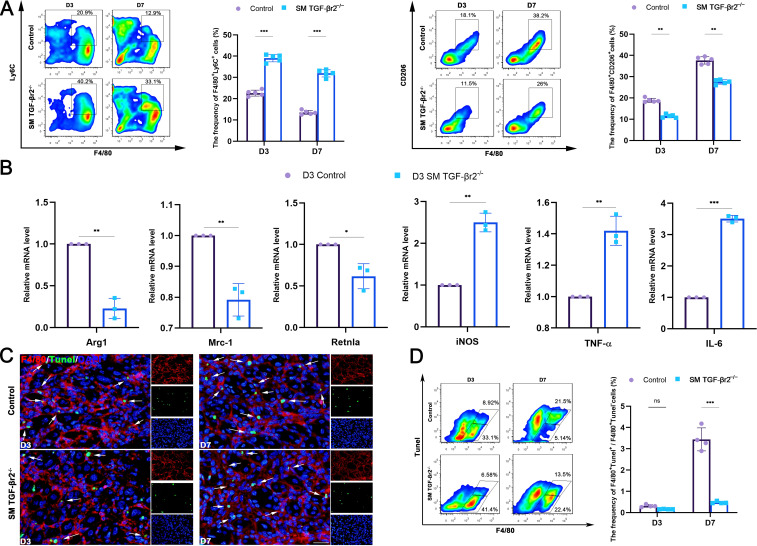
Muscle-specific TGF-β signaling regulates macrophage phenotypes and efferocytosis in inflamed muscle. **(A)** FACS analysis of the proportion of M1 (F4/80^+^Ly6C^+^) and M2 (F4/80^+^CD206^+^) macrophages. **(B)** qRT-PCR analysis of gene levels of anti-inflammatory (Arg-1, Mrc1, Retlna) and pro-inflammatory (iNOS, TNF-α, IL-6) molecules in macrophages sorted from damaged muscle of control and SM TGF-βr2^-/-^ mice on day 3 post-myoinjury(*n* = 3). **(C)** Immunofluorescence analysis of the efferocytic macrophages (Tunel^+^F4/80^+^) and the free macrophages (Tunel^-^F4/80^+^). White arrow indicates the efferocytic macrophages. **(D)** FACS analysis of the proportion of F4/80^+^Tunel^+^, and F4/80^+^Tunel^-^ macrophages. Multiple comparisons are analyzed by One-way ANOVA. Data are presented as mean ± SD (*n* = 4). *p < 0.05, **p < 0.01, ***p < 0.001. Scale bar=50 μm. ns, not significant.

We previously demonstrated that TGF-β signaling blockade results in increased macrophage recruitment to inflamed muscle (23, 26). In the present study, we further characterized the phenotypes of intramuscular macrophages. On day 3 and 7 post-injury, inflamed muscles of SM TGF-βr2^−/−^ mice exhibited an increased percentage of pro-inflammatory M1 macrophages (F4/80^+^Ly6C^+^) and a decreased percentage of pro-resolving M2 macrophages (F4/80^+^CD206^+^) compared with control mice (**Figure 2A**). Consistently, macrophages sorted from inflamed SM TGF-βr2^−/−^ muscles on day 3 post-injury showed significantly lower mRNA levels of anti-inflammatory markers (Arg-1, Mrc1, Retlna) but higher levels of pro-inflammatory markers (iNOS, TNF-α, IL-6) than those from control mice (**Figure 2B**). These results suggest that myofiber TGF-β signaling deficiency exacerbates macrophage intramuscular accumulation but impairing their transition from an M1 to an M2 phenotype.

During the resolution phase following tissue injury, pro-resolving M2 macrophages mediate phagocytosis and clearance of apoptotic cells (27, 28). We have previously found that myofiber-specific TGF-β signaling influences macrophage efferocytosis (29). Here, we further investigated this phenomenon. Immunostaining revealed comparable numbers of TUNEL^+^ apoptotic cells in inflamed muscles of control and SM TGF-βr2^−/−^ mice on days 3 and 7 post-injury (**Figure 2C**). However, in inflamed TGF-βr2^−/−^ muscle, the ratio of apoptotic cell-associated macrophages (Tunel^+^F4/80^+^) to free macrophages (Tunel^-^F4/80^+^) was significantly reduced compared with control muscle (**Figures 2C, D**).

**Figure 3:**
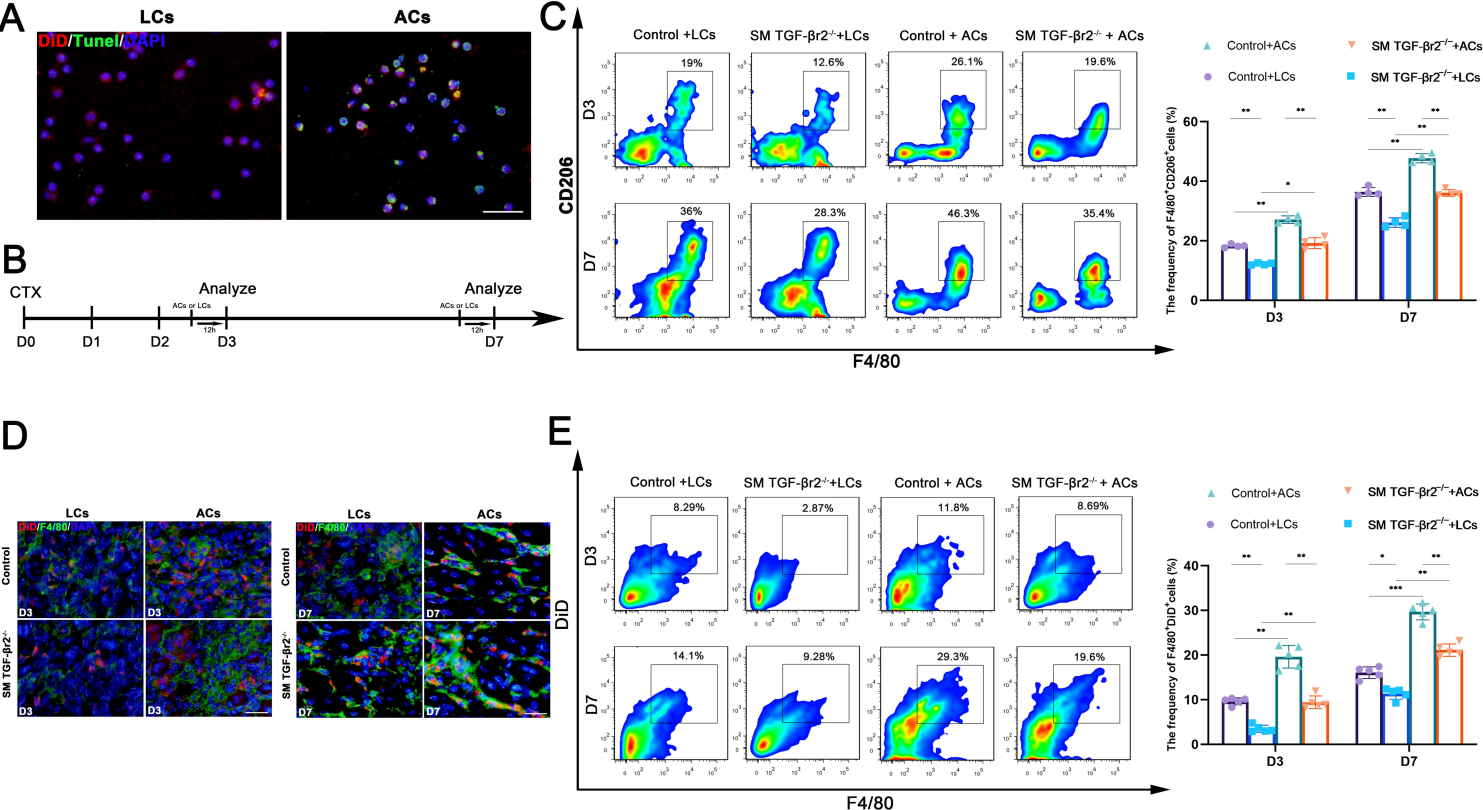
The transfer of exogenous apoptotic cells indicates muscle-specific TGF-β signaling is required for macrophages efferocytosis in inflamed muscle. **(A)** Immunofluorescence demonstrates DiD^+^Tunel^+^ apoptotic cells **(ACs)** and DiD^+^Tunel^-^ living cells (LCs). **(B)** Scheme of ACs or LCs transfer experiment. **(C)** FACS analysis of the proportion of M2 macrophages (F4/80^+^CD206^+^) in inflamed muscle after ACs or LCs transfer. **(D)** Immunofluorescence staining shows the DiD^+^ ACs uptake by macrophages in damaged muscle after ACs or LCs transfer. **(E)** FACS analysis of the proportion of the uptake of DiD^+^ ACs by macrophages. Multiple comparisons are analyzed by One-way ANOVA. Data are presented as mean ± SD (*n* = 4-5 mice per group). *p < 0.05, **p < 0.01, ***p < 0.001. Scale bar=50 μm.

To further validate the effect of muscle TGF-β signaling on macrophage efferocytosis in inflamed muscle, we generated apoptotic cells by UV irradiation and labeled them with the membrane-intercalating dye DiD (**Figure 3A**). Apoptotic cells (ACs, DiD^+^ Tunel^+^) or living cells (LCs, DiD^+^ Tunel^-^) were injected into injured TA muscles 12 hours before tissue collection (**Figure 3B**). FACS analysis showed that transfer of exogenous ACs resulted in a marked increase in the percentage of M2 macrophages (F4/80^+^CD206^+^) in inflamed muscles of control mice (**Figure 3C**). AC transfer also enhanced the uptake of DiD^+^ apoptotic cells by macrophages in control mice (**Figures 3D, E**). In contrast, in damaged muscles of SM TGF-βr2^−/−^ mice, DiD^+^ACs uptake by macrophages was markedly impaired compared with control mice, despite ACs transfer leading to an increase in M2 and efferocytosing macrophages (**Figures 3C–E**). These data indicate that myofiber-specific TGF-β signaling is required for efficient macrophage efferocytosis in inflamed muscle.

### 3.3 Muscle TGF-β signaling activation promotes macrophage IL-10-STAT3-Vav1 efferocytosis signaling by recruiting intramuscular Tregs

**Figure 4:**
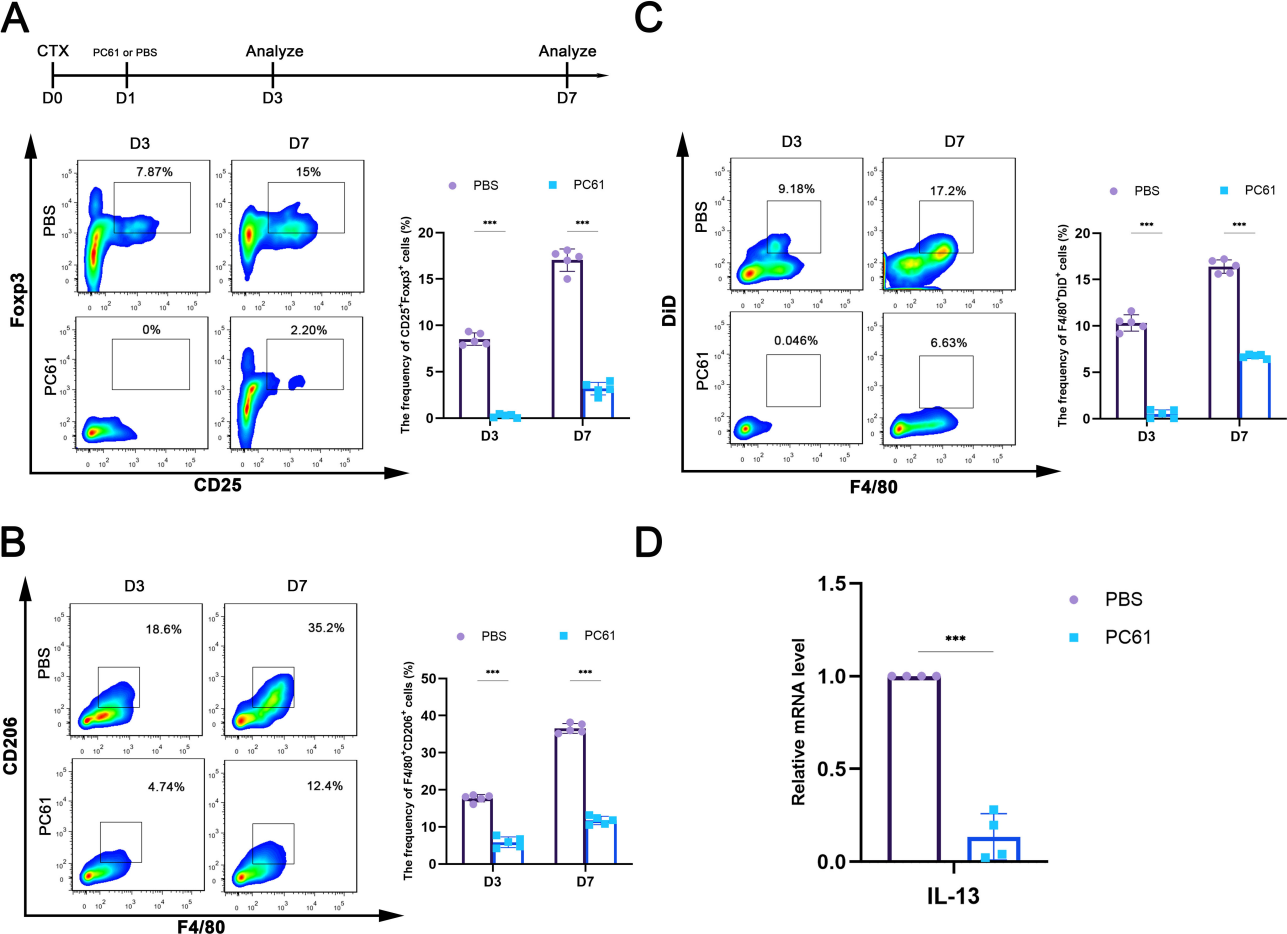
Tregs mediates macrophage efferocytosis in inflamed muscle. **(A)** Scheme of Tregs depletion using PC-61 and FACS analysis shows the depletion rate of Tregs. **(B)** FACS analysis of the proportion of M2 macrophages (F4/80^+^CD206^+^) in inflamed muscle with or without Tregs depletion. **(C)** FACS analysis of the proportion of macrophages engulfing of DiD^+^ ACs in inflamed muscle with or without Tregs depletion. **(D)** qRT-PCR analysis of IL-13 mRNA levels in Tregs sorted from inflamed muscle. Multiple comparisons are analyzed by One-way ANOVA. Data are presented as mean ± SD (*n* = 4-5 mice per group). ***p < 0.001.

It has been suggested that Tregs suppress inflammation and promote tissue repair by enhancing macrophage efferocytosis during resolution of inflammation (15). Our results have revealed a role for muscle-intrinsic TGF-β signaling in both Treg responses and macrophage efferocytosis in inflamed muscle. We therefore hypothesized that Treg-mediated macrophage efferocytosis may be involved in the muscle inflammatory process. To test this, we depleted Tregs in WT mice one day after CTX injury using anti-CD25 antibody (PC-61) (**Figure 4A**), a widely used protocol for *in vivo* Tregs depletion (30). We found that, Tregs depletion reduced the capacity of WT mice to generate M2 macrophages in inflamed muscle (**Figure 4B**). Additionally, Tregs depletion decreased the percentage of efferocytotic macrophages (F4/80^+^DiD^+^) (**Figure 4C**) and downregulated IL-13 gene level in Tregs sorted from inflamed muscle (**Figure 4D**). Given that Treg-derived IL-13 is critical for enhancing macrophage efferocytosis (15), these results imply that the intramuscular inflammatory milieu can induce Treg-associated macrophage efferocytosis.

**Figure 5:**
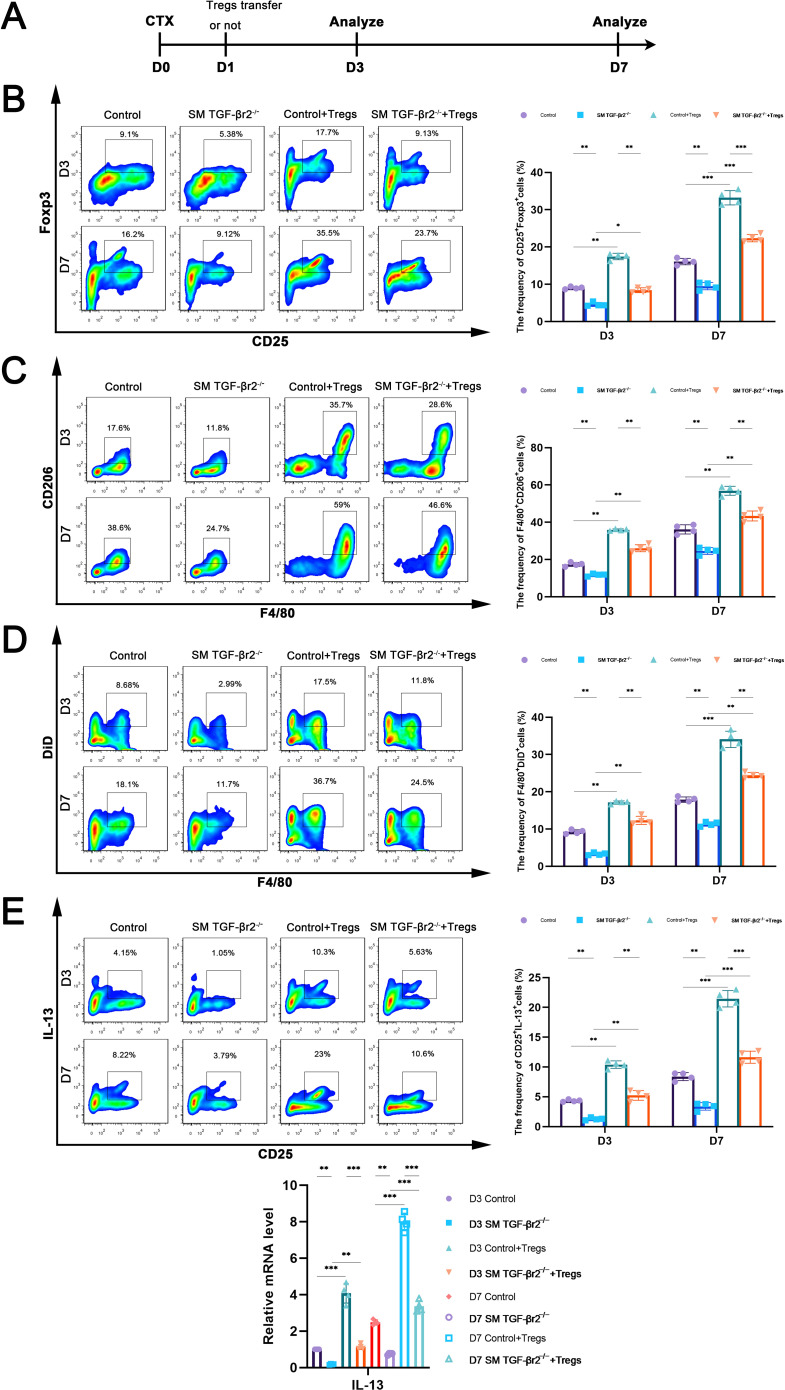
The transfer of exogenous Tregs enhances macrophage efferocytosis in inflamed muscles. **(A)** Scheme of exogenous Tregs transfer experiment. **(B)** FACS analysis of the proportion of CD25^+^ cells after Tregs transfer or not. **(C)** FACS analysis of M2 macrophages (F4/80^+^CD206^+^) in inflamed muscle after Tregs transfer or not. **(D)** FACS analysis of the proportion of macrophages uptaking of DiD^+^ ACs in inflamed muscle after Tregs transfer or not. **(E)** FACS and qRT-PCR analysis of IL-13 protein and mRNA levels after Tregs transfer or not. Multiple comparisons are analyzed by One-way ANOVA. Data are presented as mean ± SD (*n* = 4). *p < 0.05, **p < 0.01, ***p < 0.001.

Having observed a reduced population of Foxp3^+^ Tregs in inflamed muscle lacking of TGF-β signaling, we next sought to determine whether muscle TGF-β signaling acts on Treg-associated macrophage efferocytosis in injured muscle. We sorted CD4^+^CD25^+^ Tregs from WT mouse spleen by FACS and transferred these exogenous Tregs into inflamed muscles of SM TGF-βr2^−/−^ or control mice one day after CTX treatment (**Figure 5A**). Exogenous Treg transfer increased the number of CD25^+^ Tregs and F4/80^+^CD206^+^ M2 macrophages in both SM TGF-βr2^−/−^ and control recipient mice on days 3 and 7 post-injury (**Figures 5B, C**). However, significantly fewer M2 macrophages were detected in inflamed muscles of SM TGF-βr2^−/−^ mice compared with controls (**Figure 5C**). Importantly, when received co-transfer of exogenous Tregs and apoptotic cells, we monitored the fewer DiD^+^ macrophages (**Figure 5D**) in inflamed muscle and lower IL-13 gene level in Tregs sorted from SM TGF-βr2^−/−^ mice compared with controls (**Figure 5E**). These data suggest that, muscle TGF-β signaling activation contributes to Treg-mediated enhancement of macrophage efferocytosis in inflamed muscle after myoinjury.

**Figure 6:**
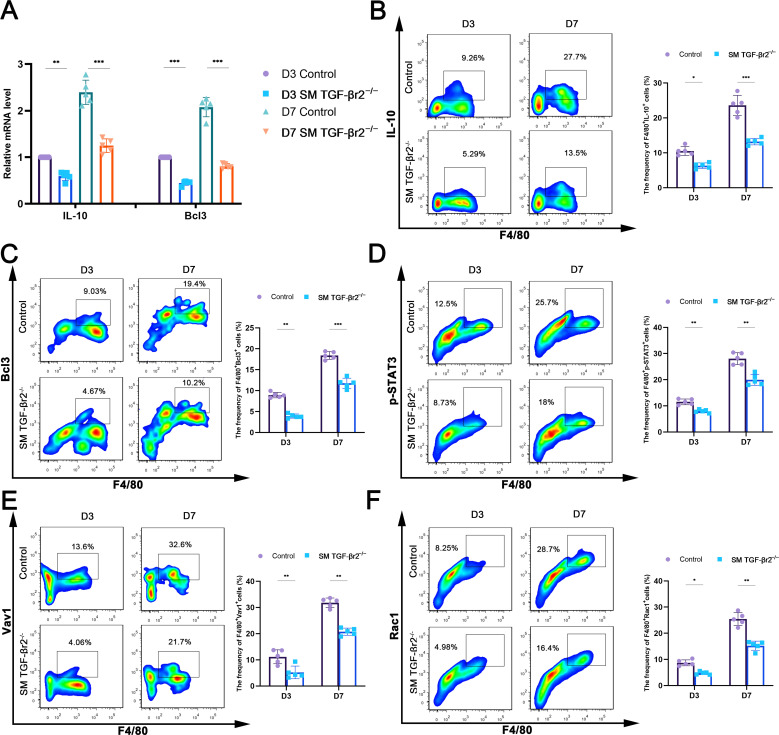
Muscle TGF-β signaling regulates IL-10-STAT3-Vav1 efferocytosis signaling in macrophages. **(A)** qRT-PCR analysis of IL-10 and Bcl3 mRNA levels in macrophages sorted from the damaged muscle. FACS analysis of the proportion of macrophages expressing IL-10 **(B)**, Bcl3 **(C)**, p-STAT3 **(D)**, Vav1 **(E)** or Rac1 **(F)** in damaged muscle respectively. Multiple comparisons are analyzed by One-way ANOVA. Data are presented as mean ± SD (*n* = 5). *p < 0.05, **p < 0.01,*** *p* < 0.001.

Treg-derived IL-13 stimulates macrophage efferocytosis *via* an IL-10-Vav1-Rac1 apoptotic cell internalization pathway in macrophages (15). We therefore reasoned that myofiber TGF-β signaling is necessary for Treg-mediated, IL-10-dependent Rac1 activation and efferocytosis enhancement in macrophages within inflamed muscle. PCR analysis revealed lower mRNA levels of IL-10 and its target gene Bcl3 in macrophages sorted from inflamed muscles of SM TGF-βr2^−/−^ mice compared with controls (**Figure 6A**). FACS analysis also showed reduced percentages of macrophages expressing IL-10 and Bcl3 (**Figures 6B, C**), as well as decreased proportions of F4/80^+^p-STAT3^+^, F4/80^+^Vav1^+^ and F4/80^+^Rac1^+^ cells in inflamed muscle from SM TGF-βr2^−/−^ mice compared with controls (**Figures 6D–F**). Collectively, these data suggest the existence of a Treg-driven IL-10-STAT3-Vav1 efferocytosis signaling pathway in muscle macrophages, which is operated by a myofiber TGF-β signaling-dependent mechanism following muscle injury.

### 3.4 Intrinsic TGF-β signaling activation suppresses myofiber IL-6 production, thereby promoting Treg-mediated macrophage efferocytosis in inflamed muscle

**Figure 7:**
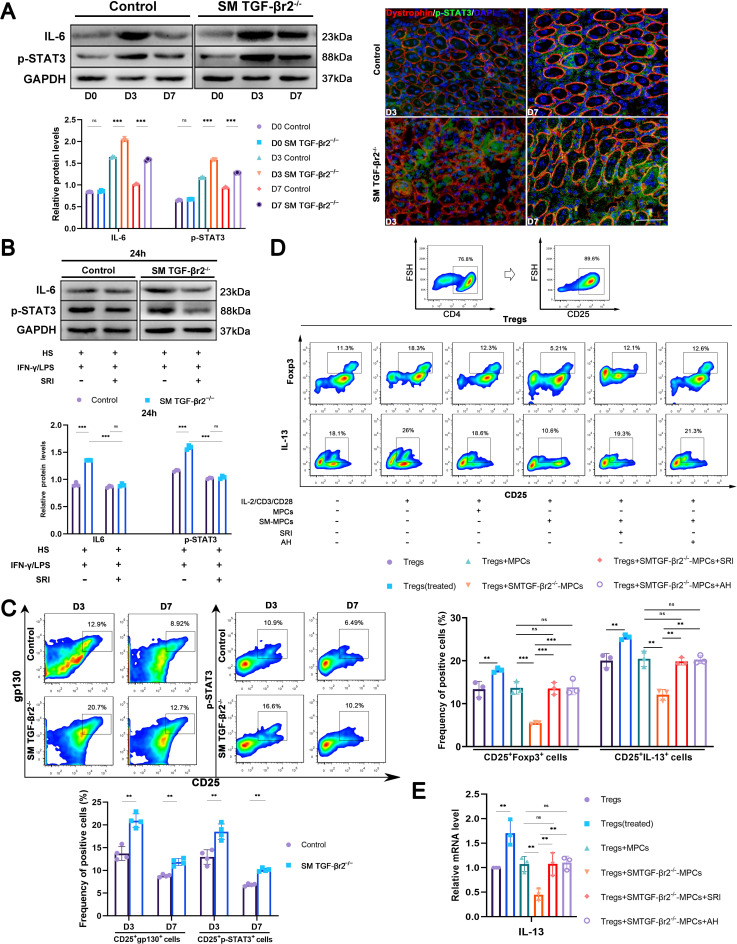
Muscle TGF-β signaling prompts to Tregs-mediated macrophage efferocytosis by suppressing myofiber IL-6 production. **(A)** Western blot and immunofluorescence analysis show IL-6 and p-STAT3 expression in inflamed muscle (*n* = 3). **(B)** Western blot analysis of IL-6 and p-STAT3 protein levels in control or SM TGF-βr2^-/-^ MPC-myotubes cultured in pro-inflammatory milieu, with or without SRI administration (*n* = 3). **(C)** FACS analysis of the proportion of CD25^+^p-STAT3^+^ cells and CD25^+^gp130^+^ cells in inflamed muscle (*n* = 4). **(D)** FACS analysis of the proportion of CD25^+^Foxp3^+^ cells and CD25^+^IL-13^+^ cells in Tregs co-cultured with TGF-βr2^-/-^ or control-MPC-myotubes, treated with or without AH or SRI (*n* = 3). **(E)** qRT-PCR analysis of Foxp3 and IL-13 mRNA levels in Tregs co-cultured with TGF-βr2^-/-^ or control-MPC-myotubes, treated with or without AH or SRI (*n* = 3). Multiple comparisons are analyzed by One-way ANOVA. Data are presented as mean ± SD. *p < 0.05, **p < 0.01, ***p < 0.001. Scale bar=50 μm. ns, not significant.

We recently observed that inflammatory stimulation resulted in the markedly elevated mRNA and protein levels of several myokines in differentiated myogenic precursor cells (MPC-myotubes) derived from SM TGF-βr2^−/−^ mice compared with control MPC-myotubes. Among these, the increase in IL-6 expression was particularly prominent (23). Given accumulating evidence that IL-6 suppresses Foxp3 and other core Treg signature genes through transcriptional and post-transcriptional mechanisms (31, 32), we now focused on this molecule. Compared with controls, we observed higher protein levels of IL-6 and the IL-6 pathway molecule p-STAT3 in inflamed muscles of SM TGF-βr2^−/−^ mice (**Figure 7A**) and in TGF-βr2^−/−^ MPC-myotubes *in vitro* (**Figure 7B**). Immunostaining further revealed the elevated p-STAT3 expression in centrally nucleated myofibers of TGF-βr2^−/−^ muscle after injury compared with control muscle (**Figure 7A**). Treatment with the TGF-β signaling agonist SRI corrected the elevated expression of IL-6 and p-STAT3 in TGF-βr2^−/−^ MPC-myotubes (**Figure 7B**). Additionally, we observed increased percentages of CD25^+^p-STAT3^+^ and CD25^+^gp130^+^ cells in inflamed muscles of SM TGF-βr2^−/−^ mice compared with controls (**Figure 7C**). Since IL-6/gp130/STAT3 signaling has been clearly identified as a major pathway that disrupts Treg cell identity (32, 33), we hypothesized that TGF-βr2^−/−^myofibers may impair intramuscular Treg-macrophage efferocytosis responses through upregulation of myokine IL-6 production. To further validate whether myofiber TGF-β signaling-correlated IL-6 elevation affects Treg-enhanced macrophage efferocytosis in inflamed muscle, we employed an *in vitro* co-culture model. MPCs were collected from neonatal SM TGF-βr2^−/−^ or control mice and cultured in a pro-inflammatory milieu containing IFN-γ and LPS, with 2% horse serum to induce myotube formation. After 48 hours, MPC-myotubes were thoroughly rinsed and co-cultured for 4 hours with Tregs (CD4^+^CD25^+^) sorted from WT mouse spleen. *In vitro*, CD25^+^ Tregs stimulated with IL-2, anti-CD3 and anti-CD28 showed increased Foxp3 and IL-13 mRNA and protein levels (**Figures 7D, E**). However, this upregulation was blocked when Tregs were co-cultured with TGF-βr2^−/−^ MPC-myotubes under the same stimulatory conditions (**Figures 7D, E**). Notably, Foxp3 and IL-13 levels were markedly restored by adding either the IL-6 inhibitor AH or the Smad agonist SRI to the co-culture system (**Figures 7D, E**). These combined data support a mechanism in which myofibers modulate Tregs activation and IL-13 release through intracellular TGF-β signaling-mediated regulation of IL-6 production.

**Figure 8:**
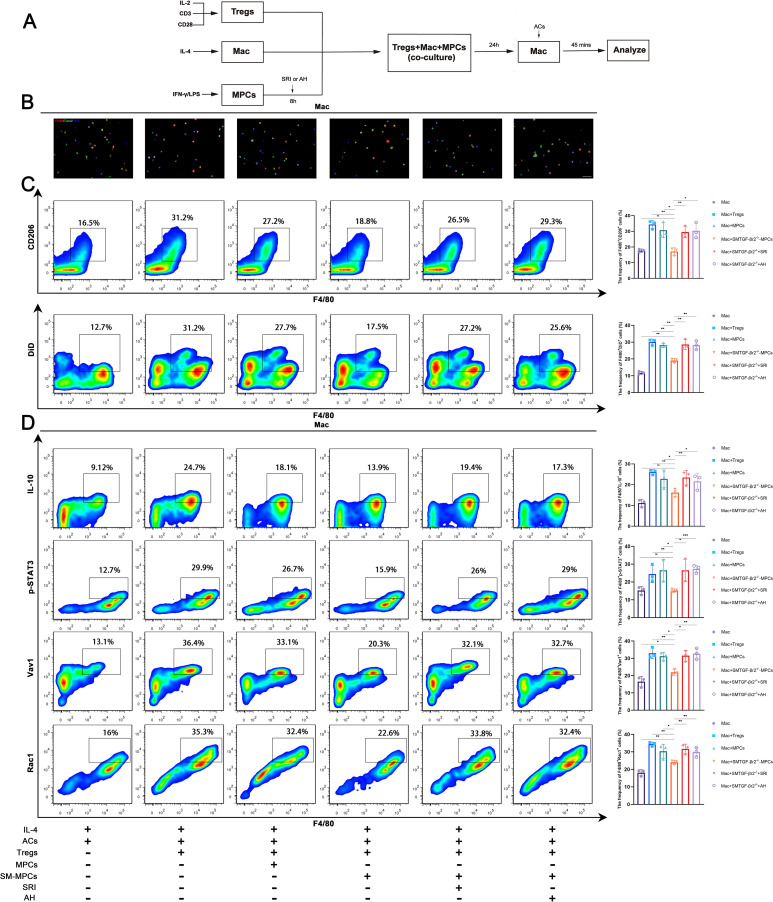
Myofiber TGF-β signaling regulates Tregs-mediated macrophage efferocytosis in vitro. **(A)** Scheme of the co-culture experiment. **(B)** Fluorescence staining shows DiD^+^ ACs engulf by macrophages in co-culture system with or without TGF-βr2^-/-^ MPC-myotubes, SRI, or AH. **(C)** FACS analysis of the proportion of F4/80^+^CD206^+^ M2 macrophages and macrophages engulfed DiD^+^ ACs in co-culture system. **(D)** FACS analysis of the proportion of F4/80^+^IL-10^+^, F4/80^+^p-STAT3^+^, F4/80^+^Vav1^+^ and F4/80^+^Rac1^+^ cells. Multiple comparisons are analyzed by One-way ANOVA. Data are presented as mean ± SD (*n* = 3). *p < 0.05, **p < 0.01, ***p < 0.001. Scale bar=50 μm.

To more definitively determine the initiating capacity of myofiber TGF-β-IL-6 signaling on Tregs-mediated enhancement of macrophage efferocytosis, we next cultured Tregs (stimulated with IL-2, anti-CD3 and anti-CD28) and macrophages (stimulated with IL-4) for approximately 4 hours. Cells were then rinsed and co-cultured for 4 hours, followed by incubation with DiD-labeled apoptotic cells (DiD^+^ ACs) for 45 minutes, in the presence or absence of TGF-βr2^−/−^ MPC-myotubes that had received 48 hours of pro-inflammatory stimulation *in vitro* (**Figure 8A**). Non-engulfed ACs were rinsed away, and macrophages were immediately fixed for analysis. Fluorescence staining and FACS analysis showed that macrophages enhanced ACs uptake in response to co-cultured Tregs (**Figures 8B, C**). However, the addition of TGF-βr2^−/−^ MPC-myotubes failed to boost macrophage efferocytosis, as evidenced by lower percentages of TUNEL^+^F4/80^+^ or DiD^+^F4/80^+^ cells in the co-culture system (**Figures 8B, C**). In contrast, adding the Smad agonist SRI or the IL-6 inhibitor AH to the assays markedly increased the percentage of efferocytotic macrophages co-cultured with Tregs (**Figures 8B, C**). These data suggest that myofiber TGF-β signaling mediates Treg-dependent macrophage efferocytosis by regulating myokine IL-6 production.

To further test this hypothesis, we examined the expression of molecules involved in the Treg-macrophage efferocytosis pathway in macrophages co-cultured with Tregs, with or without stimulation by TGF-βr2^−/−^ MPC-myotubes. The addition of TGF-βr2^−/−^ myotubes markedly reduced the expression of the pro-resolving M2 marker CD206 in IL-4-treated macrophages incubated with Tregs (**Figure 8C**) and downregulated the expression of efferocytosis-related molecules in macrophages, including IL-10, p-STAT3, Vav1, and Rac1 (**Figure 8D**). Of note, the expression of both efferocytosis and M2 markers in macrophages could be restored by the additional administration of SRI or AH (**Figures 8C, D**). In summary, our observations suggest that, in CTX-induced inflamed muscle, myofibers directly mediate Treg-dependent macrophage efferocytosis, through intrinsic TGF-β signaling induced- inhibition of myokine IL-6 production.

## 4 Discussion

Muscle represents a major constituent of the body, yet the precise mechanisms governing inflammation and repair toward myoinjury are still incompletely delineated. In this study, we examined the effects of muscle-specific TGF-β signaling on macrophage efferocytosis and Tregs response in CTX-damaged muscle. We demonstrate herein that, intrinsic TGF-β signaling is favorable for muscle regeneration, through inhibiting myofiber IL-6 production, thereby promoting Treg-mediated macrophage efferocytosis in inflamed muscle.

Resolution of inflammation through macrophage efferocytosis is essential for effective muscle regeneration. Recent studies have elucidated various molecular mechanisms regulating efferocytosis in inflamed muscle. For instance, scavenger receptor class B1 (SRB1) and the TAM receptor Mer in macrophages are required for the clearance of dead myoblasts (34, 35). The RhoA/Rac1 signaling axis regulates the transcription factor Nfix, which is involved in the expression of anti-inflammatory genes (36). The metabolic regulator AMPK, activated by the resolvin Annexin A1 through the FPR2/Axl receptor, is necessary for efferocytosis-dependent resolution of muscle inflammation (37). Resolvins D1 and D2 enhance efferocytosis and accelerate muscle regeneration (38). Soluble cytokine-mediated induction of macrophage efferocytosis also plays an important role in inflammation resolution. IL-4 and/or IL-13 coordinate anti-inflammatory phenotypes and efferocytic responses in bone marrow-derived macrophages stimulated with apoptotic neutrophils (39). The glycolytic pathway supporting efferocytosis in macrophages contributes to inflammation resolution, as conditioned medium from efferocytic macrophages induces IL-10 and TGF-β expression in naïve murine macrophages (40). A molecular network linking efferocytosis to IL-10 production *via* stimulation of mitochondrial activity has been identified in murine macrophages following myocardial infarction (41). More recently, our work revealed that reduced IL-10 production in regenerating myofibers with TGF-β signaling deficiency impairs macrophage efferocytosis in inflamed muscle (29). In recent years, it has been increasingly demonstrated that IL-10 induces the Rac1 guanine nucleotide exchange factor Vav1 in macrophages, which is necessary for enhanced Rac1 activation and efferocytosis (15). In the present study, we demonstrate that in inflamed muscle, macrophages enhance apoptotic cell internalization by activating IL-10-mediated Rac1 signaling (**Figure 6**).

Regulation of Tregs is critical for orchestrating the transition from inflammation to repair in skeletal muscle. Tregs reach peak numbers approximately 4 days after acute muscle injury, coinciding with the effective replacement of pro-inflammatory macrophages by anti-inflammatory phenotypes, a hallmark of the onset of muscle regeneration (14). Tregs facilitate muscle regeneration through multiple mechanisms: (i) stimulating muscle stem cell (MuSC) proliferation *via* the epidermal growth factor (EGF) family member amphiregulin (Areg) (42, 43)^;^ (ii) secreting Areg and TGF-β to support myofiber formation and extracellular matrix remodeling, thereby promoting tissue repair (14, 44)^;^ (iii) inhibiting conventional T cell (Tconv) activation and proliferation by consuming IL-2 and expressing CD39 and CD73, which catalyze the degradation of nucleotides such as ATP into adenosine-a metabolite that suppresses activated Tconv cells *via* A2A receptor binding, thereby reducing inflammation (45)^;^ and (iv) controlling macrophage phenotypic switching, including suppressing pro-inflammatory macrophages and promoting anti-inflammatory macrophages (15, 46). we demonstrate that in the CTX-induced inflamed muscle niche, CD4^+^CD25^+^Foxp3^+^ Tregs accumulate and exhibit elevated expression of the activation markers CTLA-4 and ICOS (**Figure 1**). Treg depletion reduces the capacity of WT mice to generate M2 macrophages and decreases the percentage of efferocytic macrophages in inflamed muscle (**Figure 4**), suggesting that Treg-associated macrophage efferocytosis operates in damaged muscle. Treg-mediated enhancement of macrophage efferocytosis has been previously reported in models of zymosan-induced peritonitis and lipopolysaccharide-induced lung injury (15). Mechanistic studies revealed that Tregs secrete IL-13, which stimulates IL-10 production in macrophages, subsequently inducing Vav1 and activating Rac1 to promote apoptotic cell engulfment (15). Our *in vivo* experiments involving Treg depletion and co-transfer of Tregs with apoptotic cells in damaged muscle (**Figure 4**, **5**) support the existence of a Treg-driven IL-10-STAT3-Vav1 efferocytosis signaling pathway in macrophages that promotes muscle regeneration.

IL-6, a pleiotropic cytokine, is required for myogenic differentiation and inflammatory responses following muscle injury. In response to muscle damage, muscle cells as well as infiltrating lymphocytes and endothelial cells secrete IL-6, which regulates immune cell function during tissue repair (47–49). IL-6 is a known suppressor of Foxp3 and other core Treg signature genes. The IL-6/STAT3 pathway has been implicated in dismantling Treg cell identity (31). Together with TGF-β, IL-6 promotes Th17 cell differentiation while suppressing Foxp3 function; IL-6 downregulates Foxp3 binding to chromatin in the presence of TGF-β (31). We previously observed myofiber-derived IL-6 production in CTX-injured muscle and in cultured primary MPCs subjected to pro-inflammatory stimulation (23). Furthermore, we demonstrated that muscle TGF-β signaling modulates myokine IL-6 production and directs muscle-specific Treg cell responses in inflamed muscle (23). In the current study, we further show that deficiency of TGF-β signaling in muscle results in markedly elevated protein levels of IL-6 and p-STAT3 in myofibers, along with increased percentages of CD25^+^p-STAT3^+^ and CD25^+^gp130^+^ cells in inflamed muscle (**Figures 7A–C**), suggesting that TGF-β signaling may cooperate with muscle cell-specific induction of IL-6. *In vitro*, co-culture with TGF-βr2^-/-^ myotubes inhibited Treg cell expression of Foxp3 and IL-13 (**Figures 7D, E**). Finally, we demonstrate that the addition of TGF-βr2^-/-^ myotubes impaired apoptotic cell uptake and reduced the expression of efferocytosis-related molecules and M2 markers in macrophages co-cultured with Tregs (**Figure 8**).

**Figure 9:**
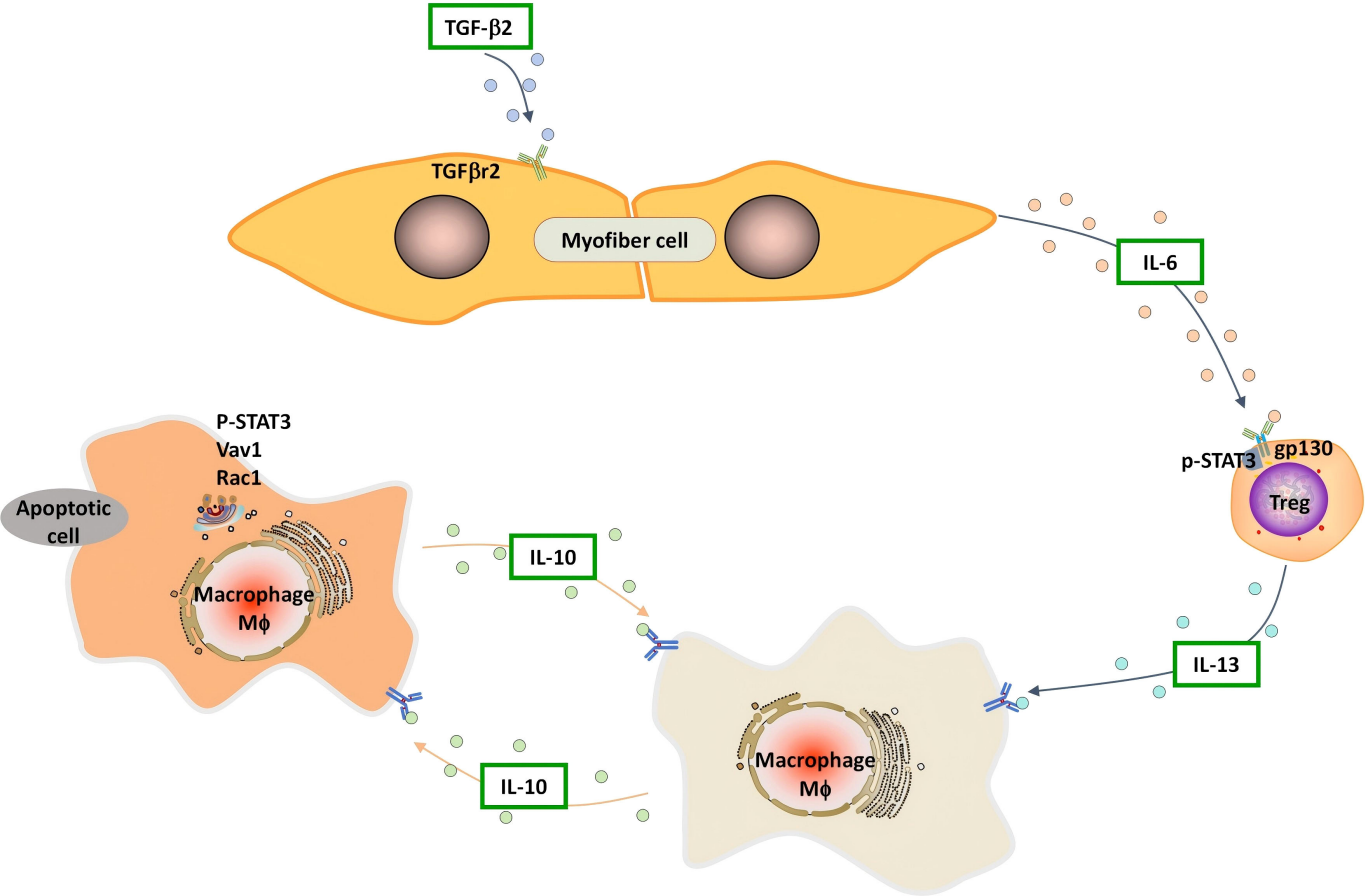
Proposed model depicting the mechanism by which myofibers mediate Treg-dependent macrophage efferocytosis through its intrinsic TGF-β signaling inhibit IL-6 production.

Collectively, our findings suggest a mechanistic link among myofiber-specific TGF-β signaling dependent-IL-6 production, Tregs derived-IL-13, IL-10-STAT3-Vav1-mediated macrophage efferocytosis in inflamed muscle (**Figure 9**). These insights raise the possibility that therapeutic targeting of this axis may promote muscle regeneration.

## Data Availability

The original contributions presented in the study are included in the article/**Supplementary Material**. Further inquiries can be directed to the corresponding authors.
